# Acute idiopathic spinal subdural hematoma: a case report

**DOI:** 10.1093/jscr/rjaf230

**Published:** 2025-04-28

**Authors:** David Shaked Zari, Ronen Spierer, Itay Ron, Jacob Shapira, Doron Norman, Shadi Hayek, Wassim Mazarieb

**Affiliations:** Technion Israel Institute of Technology, The Ruth and Bruce Rappaport Faculty of Medicine, Haifa, Israel; Rambam Health Care Campus, Haifa District, Israel; Technion Israel Institute of Technology, The Ruth and Bruce Rappaport Faculty of Medicine, Haifa, Israel; Technion Israel Institute of Technology, The Ruth and Bruce Rappaport Faculty of Medicine, Haifa, Israel; Technion Israel Institute of Technology, The Ruth and Bruce Rappaport Faculty of Medicine, Haifa, Israel; Rambam Health Care Campus, Haifa District, Israel; Technion Israel Institute of Technology, The Ruth and Bruce Rappaport Faculty of Medicine, Haifa, Israel; Rambam Health Care Campus, Haifa District, Israel; Technion Israel Institute of Technology, The Ruth and Bruce Rappaport Faculty of Medicine, Haifa, Israel; Rambam Health Care Campus, Haifa District, Israel; Technion Israel Institute of Technology, The Ruth and Bruce Rappaport Faculty of Medicine, Haifa, Israel; Rambam Health Care Campus, Haifa District, Israel

**Keywords:** spinal subdural hematoma, spine surgery, neurology, neuroradiology

## Abstract

Acute idiopathic (spontaneous) spinal subdural hematoma (spinal subdural hematomas is a rare neurosurgical emergency, often linked to coagulation disorders, trauma, iatrogenic, and underlying neoplasm, with symptoms worsening gradually. A healthy 34-year-old woman developed sudden paraplegia after back pain. Urgent T1–T2 laminectomy revealed a subdural hematoma, leading to successful evacuation. Eight months postoperative, she walks independently with reduced spasticity and intact muscle strength. The initial presentation may be subtle and symptoms worsen rapidly. Magnetic resonance imaging is the best diagnostic tool, and early decompressive laminectomy is crucial if needed.

## Introduction

Spinal subdural hematomas (SDHs) are rare and occur less frequently than intracranial subdural or spinal epidural hematomas. The primary causes of spinal SDHs include posttraumatic injuries, anticoagulant use, coagulopathy, vascular malformations, infections iatrogenic factors (such as those following surgery or lumbar puncture), or underlying neoplasms [1, 2]. In some cases, no identifiable cause is found, classifying them as idiopathic (spontaneous) spinal SDH, an even rarer condition. The largest review in the literature includes 127 cases. Therefore, we present the management of a previously healthy young woman with acute idiopathic spinal SDH.

## Case report

**Figure 1:**
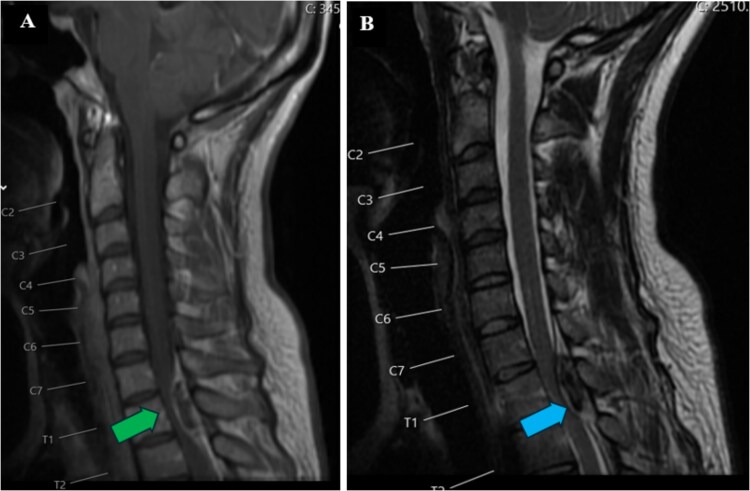
Preoperative MRI of the cervical spine. T1 (A) and T2 (B) sagittal views. Lesion is hyperintense (green arrow) on T1-weighted image and hypointense to isointense (blue arrow) on T2-weighted image at C7-T2 level.

A 34-year-old female with no significant medical history and no regular medications presented to the emergency room with sudden onset of lower cervical to upper thoracic back pain radiating to the left shoulder. The symptoms rapidly progressed, leading to the loss of independent walking despite no prior trauma. Within hours, spastic paraplegia with sensory loss below the T1 level bilaterally developed. Initial blood tests were normal, including a complete blood count and coagulation profile. Total neuroaxis CT and thoracic spine CTA revealed a mass occupying the posterior aspect of the spinal canal between C7 and T2. Subsequent magnetic resonance imaging (MRI) indicated findings consistent with an acute spinal subdural hematoma causing cord compression, most pronounced at the C7-T2 level, without any changes in cord signal as shown in Fig. 1.

An urgent T1–2 decompressive laminectomy revealed a subdural hematoma with no pathological vessels. Afterward, the intact dura was carefully opened with a dura scalpel, exposing the well-organized hematoma. The laminectomy was extended (partial C7, partial T2) to fully access the hematoma and inspect for pathological vessels. The hematoma was completely evacuated, relieving pressure without complications. The dura was then sutured, and synthetic dural replacement material with biological glue was applied.

**Figure 2:**
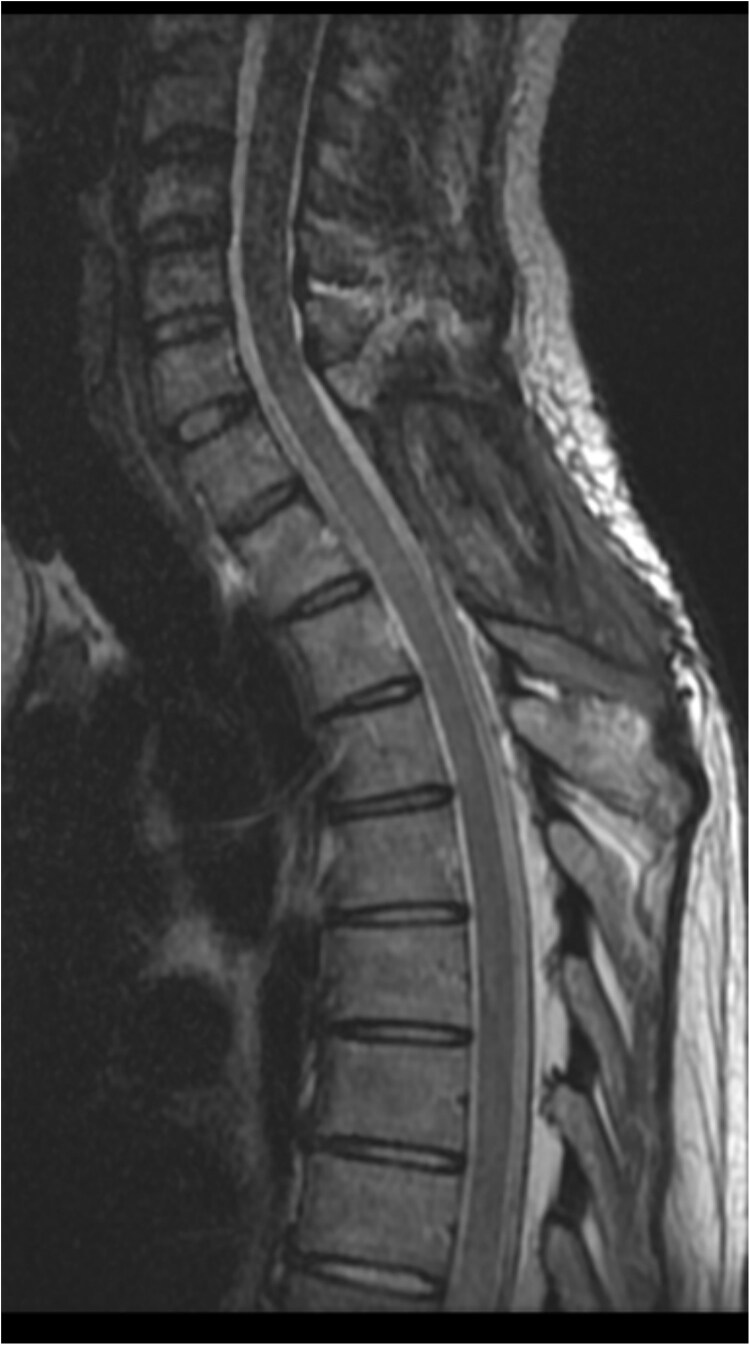
Eight months postoperative T2-weighted sagittal views MRI of the cervical spine.

The postoperative plan included early mobilization, sensory and balance training, progressive gait exercises, and follow-up imaging (DSA or MRA) to rule out missed pathological vessels. Neurological function improved, with motor strength rated 4/5 in the left leg and 3/5 in the right. Postoperative imaging was satisfactory, with no evidence of spinal cord compression as shown in Fig. 2. The patient was advised to continue occupational therapy for lower limb strengthening and physiotherapy to ensure safe ambulation.

## Discussion

Spinal SDHs are rare, with the first documented case reported by Duverney in 1682. These hematomas can cause significant neurological deficits, and risk factors include anticoagulant use, coagulopathy, vascular malformations, infections, and procedures. In some cases, no identifiable cause is found, classifying them as idiopathic spinal SDH, which is even rarer [1].

The time from hemorrhage onset to clinical presentations ranges from a few hours to several days, with a mean time of approximately 72 hours [2]. MRI is the diagnostic tool of choice. Treatment generally involves decompressive laminectomy and hematoma evacuation, which have been reported in 72% of the cases [3]. However, conservative management may be considered for patients with minimal neurological deficits or poor general health [4]. The condition has a mortality rate of 1.3% and morbidity of 28% [5].

De Beer *et al.* [3] conducted the largest review of idiopathic spinal SDHs, analyzing 122 cases with a mean patient age of 59 years. Coagulation abnormalities were identified in 48% and no underlying cause in 43%.The thoracic spine was the most frequently affected region, followed by the cervicothoracic and thoracolumbar. Common symptoms included paraparesis or paraplegia, sensory deficits, and pain. Notably, 6% of patients experienced radiculating pain without focal neurological deficits. Furthermore, 59% of patients had a favorable outcome [3].

Pathogenesis remains unclear, but a widely accepted theory suggests it is caused by subarachnoid hemorrhages. The rich capillary network and large radiculomedullary vessels in the subarachnoid space are particularly prone to rupture under increased intra-abdominal or intrathoracic pressure, such as from yawning, coughing, or minor trauma. Initially, the blood is diluted and redistributed by cerebrospinal fluid (CSF) flow, preventing clot formation. As bleeding continues, larger clots may form, obstructing CSF flow and leading to further clot accumulation. This stagnation in CSF flow contributes to further clot accumulation in the subarachnoid space. Eventually, this leads to the rupture of the arachnoid membrane and causes SDH [6, 7]. Another less common theory proposes that spinal SDH results from the migration of an intracranial SDH, driven by the combined effects of bridge vein rupture, anatomical continuity, and gravitational forces [8–10]. Clinical relevance- Idiopathic spinal SDH presents a neurological emergency. The initial presentation may be subtle and nonspecific, such as mild back pain with symptoms worsening rapidly within hours. MRI is the best imaging method to diagnose and if necessary decompressive laminectomy should be done as soon as possible.
